# Effect of Ascorbic Acid on Shear Bond Strength of Orthodontic Brackets Bonded with Resin-modified Glass-ionomer Cement to Bleached Teeth

**DOI:** 10.5681/joddd.2012.013

**Published:** 2012-06-06

**Authors:** Behnam Khosravanifard, Vahid Rakhshan, Solmaz Araghi, Hadi Parhiz

**Affiliations:** ^1^Associate Professor, Department of Orthodontics, Dental Branch, Islamic Azad University, Tehran, Iran; ^2^Academic Staff and Lecturer, Department of Dental Anatomy and Morphology, Dental Branch, Islamic Azad University, Tehran, Iran; ^3^Postgraduate Student, Department of Endodontics, School of Dentistry, Qazvin University of Medical Sciences, Qazvin, Iran; ^4^Postgraduate Student, Department of Orthodontics, Dental Branch, Islamic Azad University, Tehran, Iran

**Keywords:** Ascorbic acid, orthodontic brackets, tooth bleaching, resin-modified glass-ionomer, shear bond strength (SBS)

## Abstract

**Background and aims:**

Bleaching can considerably reduce shear bond strength (SBS) of orthodontic brackets bonded with composite adhesives. Application of antioxidants is a method to reverse the negative effect of bleaching on composite-to-enamel bond. However, the efficacy of antioxidants in increasing the SBS of brackets bonded using resin-modified glass-ionomer cement (RMGIC) has not been studied, which was the aim of this study.

**Materials and methods:**

Fifty freshly extracted human maxillary first premolars were bleached with 35% hydrogen peroxide (Pola Office Bleaching, SDI). Sodium ascorbate 10% was applied to the experimental specimens (n=25). All the specimens were etched with 37% phosphoric acid (Ivoclar/Vivadent) and bonded using RMGIC (Fuji Ortho LC, GC). The specimens were subjected to incubation (37°C, 24h) and thermocycling (1000 cycles, 5-55°C, dwell time = 1 min). The SBS was measured at 0.5 mm/min debonding crosshead speed. The adhesive remnant index (ARI) was scored under ×10 magni-fication. Data were analyzed using Mann-Whitney U test, one- and independent-samples t-test, and Fisher’sexact test (α=0.05).

**Results:**

The mean SBS of experimental and control groups were 11.97 ± 4.49 and 7.7 ± 3.19 MPa, respectively. The dif-ference was statistically significant (P=0.000 by t-test). SBS of both control (P=0.014) and experimental (P=0.000) groups were significantly higher than the minimum acceptable SBS of 6 MPa, according to one-sample t-test.

**Conclusion:**

Application of ascorbic acid can guarantee a strong bond when RMGIC is to be used. However, RMGIC might tolerate the negative effect of bleaching with minimum SA treatments (or perhaps without treatments), which de-serves further studies.

## Introduction

Various bleaching agents and techniques have been advocated as safe yet effective methods to whiten discolored teeth at the office or home.^1^ Although certain studies have not reported any adverse effect of bleaching on the shear bond strength (SBS) of orthodontic brackets,^2^ some others have reported a considerable reduction in SBS levels subsequent to bleaching.^18^ The reduced SBS might be attributable to alterations in the microstructure of bleached enamel surfaces after becoming acid-etched, including reduced microhardness, calcium loss, becoming overetched, and loss of enamel prisms.^1^ In addition, other underlying mechanisms such as residual components of bleaching agents, which might release oxygen radicals on the enamel surface, have been suggested.^7- 9^ In order to avoid bonding failure (which is defined as SBS values less than 6 to 10 MPa in vitro) ^10, 14^ on bleached teeth, some methods have been proposed. These consist of avoiding the bleaching procedure until the completion of orthodontic treatment,^1,1^ delaying bracket bonding from 24 hours to 4 weeks after bleaching,^1- 5, 7 , 8 , 15^ pumicing the bleached teeth,^7 , 8^ and applying antioxidant agents prior to bracket bonding, in order to neutralize the effect of released oxygen radicals from residual bleaching components.^1 , 5 , 7- 9 , 15^19

Sodium ascorbate (SA) is a cheap and commonly available antioxidant material. Many authors have evaluated its effect on the bleached teeth when composite resins have been used as bonding adhesives.^7- 9,15, 19^ In 2002, Lai et al.^18^ asserted that when an antioxidant such as SA was applied for 3 hours to enamel after bleaching with carbamide peroxide, the composite SBS was improved. ^7 , 18^ Bulut et al ^7 , 8^ in 2005 and 2006 studied the efficacy of delayed bonding and antioxidant application and reported that both approaches were effective in returning the compromised bond strength back to the control levels. In 2008, Kaya et al.^17^ studied SA in gel form, which proved effective for enhancing the SBS of composite resin bonded to enamel. They also determined the time appropriate for the procedure. At least 60 minutes of gel application was needed for maximum effectiveness. The SBS increased as the application period of the SA increased. They suggested that application of this gel by the patient might reduce the chair time.^17 , 15^ in 2008 studied SA application and delayed bonding techniques. They reported that only the antioxidant application was effective and delayed bonding method failed to increase the SBS sufficiently. In 2009, Sasaki et al.^19^ compared the efficacy of two different antioxidizing agents in increasing the SBS of bleached enamel and dentin, reporting that 10% α-tocopherol was successful, while 10% SA was not. In 2011, Lima et al.^20^ showed that even short durations of 10% SA application (i.e., one minute) could still obviate the detrimental effect of bleaching on SBS.

Although residual bleaching materials and delayed oxygen release on the enamel surface of bleached teeth are not the only factors to decrease SBS, most of the studies on ascorbic acid have shown that SBS improves sufficiently when bonding adhesives are composite resins.^8 , 9 , 15 , 16^ However, composite resin is not the only available bonding adhesive; another common adhesive type is resin-modified glass-ionomer cement (RMGIC) which has fundamental differences compared to composite resins.^13^ These have become popular among orthodontists due to their advantages over composite resins, such as releasing fluoride, preventing white spot formation, being hydrophilic, and their appropriate results on SBS in areas difficult to isolate from moisture.^13 , 21 , 22^ Due to their broad use, it seems necessary to evaluate the behavior of RMGIC bonded to bleached teeth, and also to assess possible surface treatments to gain the best outcome. Nevertheless, the literature lacks any studies on this subject. Therefore, the aim of this study was to evaluate the efficacy of 10% SA solution in enhancing the SBS of metallic brackets bonded with RMGIC to bleached teeth. Moreover, the adhesive remnant index (ARI), which correlates with the extent of damage after adhesive removal procedures and prevalence of caries,^14^ was estimated.^8^

## Materials and Methods

This in vitro experimental study was performed on 50 intact human maxillary first premolars extracted only for therapeutic reasons from 25 orthodontic patients (with the treatment plan of bi-premolar extraction) who had given written consent forms. The specimens were sequentially approved according to the exclusion criteria which comprised the existence of hypoplasia, hypocalcification, caries on buccal surface, history of the application of previous chemical agents, and enamel fractures.^12 , 14^ To exclude the biologic or environmental confounders influencing enamel development, in case one of a patient’s premolars met the exclusion criteria, both would be excluded as well.

###  Sample Preparation

After extraction, the teeth were stored in deionized distilled water under aseptic conditions.^12^14 When 25 pairs of premolar teeth were collected, the specimens were randomly divided into a control group (without the application of antioxidant material) and an experimental group (Table 1). Each group included a random tooth from all patients; therefore, the teeth in the two groups were matched. The specimens were stored in deionized distilled water until the planned sample size was achieved, for no more than 6 months.

**Table 1:** The experimental procedures

Group	Bleaching	SA	Etching	Bracket bonding	Incubation	Thermocycling	SBS testing	ARI testing
Control	•	–	•	•	•	•	•	•
Experimental	•	•	•	•	•	•	•	•

SA, sodium ascorbate application; Bullet, present; Hyphen, absent.

**Table 2:** Descriptive statistics of shear bond strength

Group	Mean ± SD (MPa)	Min (MPa)	Max (MPa)	CV (%)	Mean 95% CI	P_6_	P_8_	P_10_
					Lower	Upper			
Control	7.7 ± 3.19	4	14.5	41.4	6.63	8.87	0.014	0.642	0.001
Experimental	11.97 ± 4.49	7	25	37.5	10.46	13.48	0.000	0.000	0.038

P_6_, P_8, _and P_10_ respectively indicate the P values calculated (using the t-test) by comparing the groups’ means with the SBS values 6, 8, and 10 MPa

### Bleaching

The specimens in both the control and experimental groups were bleached as follows. The buccal surface of each tooth was subjected to pumice prophylaxis (with water) using a low-speed rubber cup for 10 seconds. Afterward, they were rinsed (30 s) and air-dried (15 s) with an oil-free air/water syringe. The specimens were bleached using a light-curable 35% hydrogen peroxide with a pH value of 5.5 (Pola Office Bleaching, SDI, Melbourne, Australia) according to manufacturer’s instructions; the powder and liquid in the bleaching kit were blended, and the blend was applied to the buccal surface of each tooth with a disposable brush. Then it was light-cured with a calibrated unit (Arialux Blue Point, Apadanatak, Tehran, Iran) for 30 seconds. After 3 minutes, the bleaching agent was removed and the teeth were washed (30 s) and air-dried (15 s).

### Application of Ascorbic Acid

The control group was not subjected to SA application. The buccal surfaces of the bleached experimental teeth were subjected to 10 mL of 10% SA solution for 10 minutes, applied in ten 1-minute intervals. Afterward, the enamel surface was rinsed with distilled water for 30 seconds.^8^

### Etching and Bracket Bonding

All the specimens were etched with 37% phosphoric acid solution (Ivoclar/Vivadent, Liechtenstein) for 15 seconds; then they were water-sprayed (15s) and air-sprayed (15s). RMGI cement (Fuji Ortho LC, GC America, Alsip, IL, USA) was used for bonding the brackets. After mixing the powder and liquid, the homogenized mixture was smeared on the bracket backs (Mini Dyna Lock standard size 0.018, 3M Unitek, Monrovia, CA, USA). The undercuts on the bracket backings would allow the adhesive to debond only from the tooth surface. A bracket positioning gauge was used to place the brackets on the mid-buccal surfaces of the teeth at least 4 mm away from the buccal cusp ridges, while the bracket slot was perpendicular to the tooth coronal long axis. Using a force gauge (1303 16 oz, ETM, Monrovia, CA, USA) a 300-gr compressive force was aimed at each bracket to reduce and standardize the adhesive thickness.^11 - 14^ The excess materials were carefully cleaned by a dental scaler. Furthermore, each bracket was light-cured for 40 seconds according to the manufacturer (10 seconds per side: occlusal, cervical, mesial, and distal). The intensity of the output light was calibrated in the beginning and after curing every five specimens at an intensity range instructed by the RMGIC manufacturer (400500 mW/cm ^2 ^).

### Incubation and Thermal Cycling

The teeth were incubated at 37°Cfor 24 hours. Then they were thermocycled at 1000 cycles (555°C, transfer time = 15s, dwell time = 30s).

### Measuring the SBS

The specimens were mounted by their roots in self-cured acrylic resin cylinders in a vertical position. The height of the cylinder was up to the CEJ of each tooth and their diameter was 3 cm. An orthodontic wire was used to standardize the mounting procedures, as described previously in detail.^11 , 14^

The SBS was measured by vertical shear force (Universal Testing Machine 1195, Instron, Canton, MA, USA) aimed at the occlusal sides of bracket wings, exerted at 0.5 mm/min crosshead speed, and then by dividing the force (in Newton) by bracket base surface area (in mm ^2 ^) to calculate the SBS in Megapascal (MPa) .^11- 14, 23^

Using a stereo microscope (X20, Canton Optical, Canton, NY, USA) the ARI was estimated under ×10 magnification.^8^

Descriptive statistics were calculated. After confirming the normality of SBS distribution using a Kolmogorov-Smirnov test, the SBS data were analyzed by two-tailed one- and independent-samples Student’s t-tests. The frequency distribution of SBS values in the two groups was compared using Fisher’s exact test and the attributable risk (AR) was calculated. The ARI data were analyzed using Mann-Whitney U test. The level of significance was set at 0.05.

## Results

**Figure 1:**
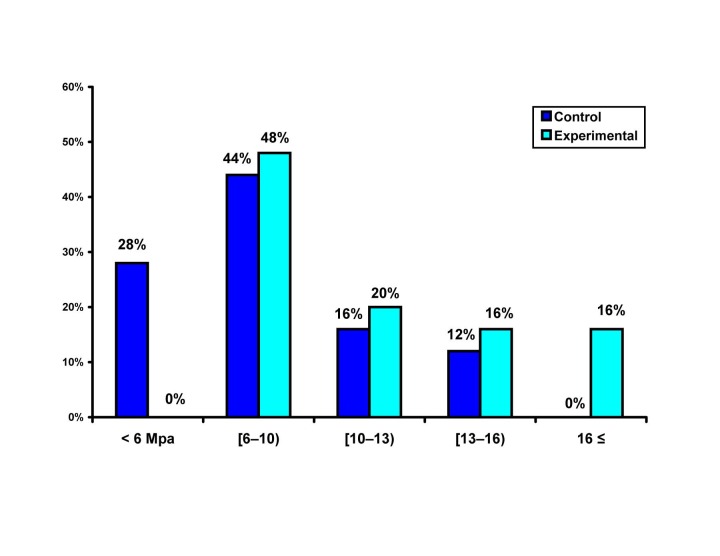
Frequency distribution (%) of SBS values (MPa).

According to one-sample t-test, the SBS of the control group was 8 MPa, being significantly different from 6 and 10 MPa values. The SBS of the experimental group was significantly greater than all the test values (Figure 1).

**Figure 2:**
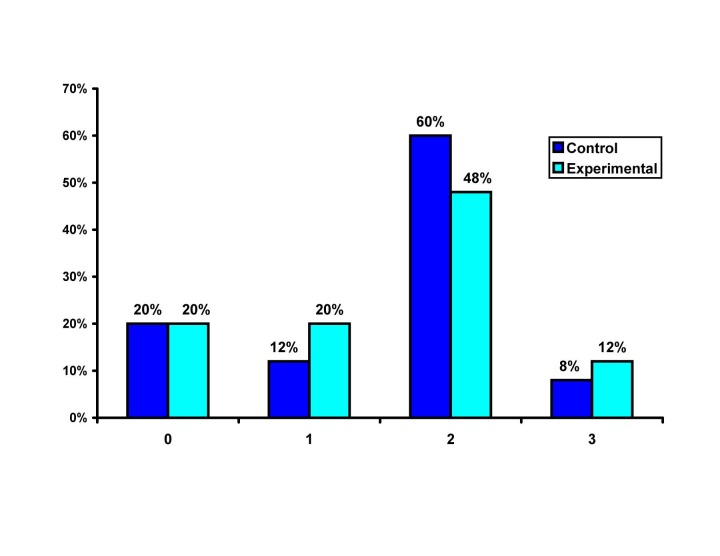
Frequency distribution (%) of ARI scores.

Comparison between the means of the two groups (Figure 2) using the independent-samples t-test showed a significantly higher SBS in the experimental group (P=0.000, 95% CI for means’ difference = 2.016.49).

Assessing the frequency distribution of specimens with bond strengths lower than 6 MPa (Figure 1) by Fisher’s exact tes, revealed a significant difference between the two groups (P=0.006). The AR was calculated to be 28%, meaning that in cases in which sodium ascorbate solution was not applied, the possibility of producing bond strengths lower than 6 MPa would increase up to 28%. Comparison of the ARI scores of the two groups (Figure 2) using Mann-Whitney U test did not show a significant difference (P=0.3).

## Discussion

There have been some controversies over the lowest acceptable SBS limit, being reported between 6 and 8 MPa or between 8 and 10 MPa.^10, 14^14 Therefore, all the three values were used in this study. Despite incubation and thermocycling procedures which might considerably reduce SBS, ^13^ the control group reached acceptable levels of SBS. Therefore, one might conclude that RMGIC could be used on bleached teeth without an earlier treatment or with the minimal necessary treatments. Cacciafesta et al^4^ reported a similar SBS rate for Fuji Ortho LC RMGIC on bleached teeth, supporting the validity of the results of this study. They reported that the SBS levels might drop from a possible base of 11.67 ± 2.4 MPa in the control group to 8.68 ± 1.78 MPa after bleaching.^4^ The presence of thermal cycling in this study and use of bovine teeth and application of a different etchant (10% acrylic acid) in that study might have contributed to the subtle differences observed.^4 , 13^

RMGIC seemed to be less sensitive to bleaching. The oxygen inhibits the polymerization of the composite resin.^21 , 22^ However, apparently the low fraction of HEMA molecules in RMGIC ^22^ might make it less vulnerable to oxygen compared with composite resins. This might confirm the studies claiming that the chemical mechanism underlying the negative effect of bleaching is a major reason.^8 , 9^ On the other hand, the application of 10% sodium ascorbate solution increased the SBS considerably,^4^ indicating that free oxygen radicals did have a considerable negative effect on the mean SBS of the control group. Other factors relating to this increase might be the acidic nature of ascorbic acid which might act as an etchant, or its catalytic characteristics.^22^ However, subsequent to the application of sodium ascorbate, SBS levels reached a level similar to the control group in a study carried out by Cacciafesta et al,^4^ which might be greater than their results if thermocycling was not performed. This might imply that the acidic antioxidant agent had mostly reversed the effect of bleaching agent, and the effect was not necessarily due to a probable etching effect of 10% ascorbic acid. The control group showed sufficient SBS levels in the current study. Therefore, the dose and duration of sodium ascorbate application might be reduced to an optimum as it has been shown that shorter durations of SA application can also be useful in composite resins.^20^ In addition, the favorable findings in the control groups suggests that RMGIC might still provide sufficient bond strength when bonding the brackets to bleached enamel (with minimum or no treatments). This, however, needs further studies, especially taking into consideration the 28% risk of SBS reduction below the acceptable limit without applying any SA treatment. The aforementioned findings might point to another advantage of RMGIC to be used in orthodontics, besides being white-spot preventive, fluoride releaser, and insensitive to saliva and being less sensitive to blood compared with composite resins.^13 , 21 , 22^ It should be kept in mind that at present various bleaching agents might be used at home, which might interfere with bonding of orthodontic brackets.^2^

Since the literature does not include any similar studies, this study was only compared with studies performed on composite resin adhesives. Most previous studies have shown increases in SBS after neutralizing the bleaching agent by antioxidizing agents.^1 , 5 , 79 , 15^18 Only few studies have failed to show appropriate results for SA.^19^ Nevertheless, in a study, another antioxidant formulation (10% α-tocopherol) still showed high efficacy.^19^ Therefore, the results of that study did not discourage the application of antioxidant agents. The failure in SA results of that study might be attributed to the brand used as well as other probable methodological shortcomings such as statistical analysis and small sample size of each group. Thus, it might be concluded that the effect of antioxidant agent was partly irrelevant to the adhesive type. However, it should be noted that in the other studies on composites, it was necessary to neutralize the effect of bleaching agents, but in this study the average SBS of the control group reached the acceptable limit as well.

Although similar to the study of Cacciafesta et al^4^ no significant difference was observed in the ARI between the two groups, other studies on composite resins have demonstrated significant adhesive voids on bleached enamel surfaces, as well as fragile resin tags in such areas.^8 , 9^ The contrasting results pertaining to the two materials might be explained in the context of hydrophobic nature of composite resins used in those studies, compared to RMGI cements.^13 , 22^

This study was limited by some factors. It was better to have another control group to bond the brackets to intact non-bleached enamel. Besides, it might have been better to have other experimental groups to test different amounts and durations of SA application. This might have allowed the authors to find the optimum duration/amount of SA application. The authors tried to increase the reliability and comparability of findings by preparing large groups, matching the teeth in the control/experimental groups to eliminate genetic and environmental factors affecting enamel structure, which was not present in any of the previous studies in the field, carefully performing standardized methods, and comparing the SBS values with three different acceptable minimum SBS levels proposed in different studies. ^10- 14^ In the present study, incubation and thermocycling were utilized to simulate the oral environment.^11, 14, 23^ Nonetheless, it was impossible to completely simulate natural masticatory forces rapidly changing in magnitude, type, and direction solely by the static shear force used in this study.^24^ Additionally, the results of a particular brand of a material cannot be necessarily generalized to other brands of the same material.^12 , 23^ Therefore, further clinical studies are needed to confirm these findings.

## Conclusion

Application of 10% SA solution to bleached teeth prior to bracket bonding with RMGIC can guarantee the resistance of the bracket in vivo. Interestingly, RMGIC might endure the negative effect of bleaching on SBS even with minimum treatments. However, further studies are necessary.
